# Reduced dispensing of prescribed antibiotics during the Covid-19 pandemic has not increased severe complications from common infections

**DOI:** 10.1186/s12889-022-12692-1

**Published:** 2022-02-08

**Authors:** Christer Norman, Mikaela Svensson, Ingrid Schmidt, Vendela S. Bergfeldt, Ragda Obeid, Anders Ternhag, Johan L. Struwe

**Affiliations:** 1 Public Health Agency of Sweden, Solna, Sweden; 2 National Board of Health and Welfare, Stockholm, Sweden; 3 Department of Medicine Solna, Karolinska Institutet, Stockholm, Sweden

**Keywords:** Antibiotics, Prescriptions, Infectious complications, Covid-19

## Abstract

**Background:**

Sweden has seen an accelerated decline in the number of dispensed antibiotic prescriptions from an already low level during the Covid-19 pandemic. This prompted us to explore whether the decrease in antibiotic prescriptions has reached a critically low level and resulted in an increase in treatment of severe complications from common infections. The aim was to study if the accelerated decrease in antibiotic sales has led to an increase in complications in outpatients with common infections.

**Method:**

A population-based nationwide registry study based on the Swedish Prescribed Drug Register and the National Patient Register.

**Results:**

The total number of dispensed antibiotic prescriptions decreased by 17% during 2020 compared to 2019. The decrease was most pronounced in younger age groups and for antibiotics targeting respiratory tract infections. The number of hospital admissions and visits to open specialist care due to pneumonia or complications related to otitis, tonsillitis, or sinusitis decreased by 4–44%. Prescriptions and numbers of visits or admissions due to urinary tract infections and skin infections remained largely unchanged compared to previous years.

**Conclusion:**

No increase in complications due to common bacterial infections could be detected despite an unprecedented decline in dispensed antibiotic prescriptions in outpatient care in 2020. The decrease in dispensed antibiotic prescriptions from pharmacies was probably primarily related to a general decrease in the incidence of respiratory infections due to the recommendations and restrictions implemented to mitigate the Covid-19 pandemic in Sweden. This in return led to fewer doctors’ visits and consequently to fewer occasions to prescribe antibiotics, be they warranted or not.

**Supplementary Information:**

The online version contains supplementary material available at 10.1186/s12889-022-12692-1.

## Background

As the Covid-19 pandemic has evolved, a number of effects on the health care systems have been reported, one of them being a reduction in the sales of prescribed antibiotics. The first cases of Covid-19 were detected in February 2020 in Sweden and there was a first and a second wave that year where a large number of people were treated in intensive care (Supplementary Figs. S1-2). In Sweden, the decline in the dispensing of antibiotic prescriptions during 2020 was the largest seen over the course of a single year in the past 20 years (Supplementary Fig. S3). In the US the number of outpatients with antibiotic prescriptions decreased by nearly 40% from January to May 2020 [1]. In the UK, the number decreased by 15% during April to August compared to 2019. However, it was found that while the number of prescriptions decreased, the “density” of prescriptions actually increased during the pandemic when adjusted for the fewer consultations in primary care [2]. A systematic review recently concluded that the total number of visits to all health care decreased by 37% in the first months of the pandemic [3]. A similar decline in doctors’ consultations has also been observed in Sweden, both in primary care as well as in orthopaedic surgery, general surgery and eye surgery [4].

It is important to monitor not only the level of antibiotic sales, but also the number of severe infections and rare complications to common infections in order to determine if the level of prescriptions in the population becomes too low. Previous studies from Sweden [5] and other countries [6, 7] before the Covid-19 pandemic found no such risks.

To determine if the accelerated decrease in antibiotic sales might have reached such a critically low level that it might impose a risk for patient safety, we performed a descriptive registry-based observational study of dispensed antibiotic prescriptions and the incidence of complicating infections requiring treatment in specialist or hospital care.

## Methods

This was a population-based nationwide registry study in which the whole population of Sweden was the study base. We compared the number of persons and persons per 100,000 inhabitants admitted to hospital or seeking specialized care for nine infectious disease diagnostic groups (Table 1) 2019 and 2020. We also examined the number of antibiotic prescriptions the same period and also two additional pre-pandemic years 2017–2018.

**Table 1:** The number of persons and persons per 100,000 inhabitants admitted to hospital or seeking specialized care per diagnostic group 2019-2020

Diagnostic group (see Supplementary Table S2)	Age	2019		2020		Change 2020, relative to 2019	
		Persons	Persons/ 100,000 Inhabitants	Persons	Persons/ 100,000 Inhabitants	Persons	Percentage change
Quinsy, etc.	0-4	35	5.8	18	3.0	-17	-48.2 (-66.5, -34.9)
	5-19	845	47.2	755	41.7	-90	-11.8 (-12, -11.5)
	20-69	3 559	55.8	2 703	42.2	-856	-24.3 (-24.8, -23.9)
	70+	310	20.6	248	16.1	-62	-21.7 (-22.9, -20.5)
	**All ages**	**4 749**	**46.2**	**3 724**	**36.0**	**-1 025**	**-22.1 (-22.5, -21.8)**
Mastoiditis	0-4	70	11.6	41	6.9	-29	-41 (-49.9, -33.6)
	5-19	73	4.1	39	2.2	-34	-47.2 (-58.5, -38.1)
	20-69	44	0.7	29	0.5	-15	-34.3 (-42.5, -27.8)
	70+	7	0.5	9	0.6	2	25.9 (9.6, 69.4)
	**All ages**	**194**	**1.9**	**118**	**1.1**	**-76**	**-39.6 (-44.3, -35.4)**
Sinusitis complications	0-4	116	19.2	50	8.4	-66	-56.6 (-69.7, -45.9)
	5-19	188	10.5	110	6.1	-78	-42.2 (-47.6, -37.4)
	20-69	587	9.2	320	5.0	-267	-45.7 (-49.2, -42.4)
	70+	147	9.8	74	4.8	-73	-50.7 (-59.6, -43.2)
	**All ages**	**1 038**	**10.1**	**554**	**5.4**	**-484**	**-47 (-49.8, -44.4)**
Meningitis and brain abscesses	0-4	36	6.0	35	5.8	-1	-2.1 (-1.9, -2)
	5-19	29	1.6	24	1.3	-5	-18.3 (-21.6, -15.5)
	20-69	282	4.4	239	3.7	-43	-15.6 (-16.4, -14.8)
	70+	131	8.7	96	6.2	-35	-28.3 (-31.3, -25.5)
	**All ages**	**478**	**4.7**	**394**	**3.8**	**-84**	**-18.2 (-18.9, -17.4)**
Pneumonia	0-4	2 737	453.8	1 139	190.3	-1 598	-58.1 (-60.7, -55.5)
	5-19	2 039	114.0	989	54.6	-1 050	-52.1 (-54.5, -49.8)
	20-69	15 740	246.6	9 336	145.7	-6 404	-40.9 (-41.4, -40.4)
	70+	29 449	1 955.6	17 947	1 166.7	-11 502	-40.3 (-40.7, -40)
	**All ages**	**49 954**	**486.0**	**29 404**	**284.0**	**-20 550**	**-41.6 (-41.9, -41.3)**
Skin-and soft tissue infections	0-4	263	43.6	198	33.1	-65	-24.1 (-25.9, -22.5)
	5-19	548	30.6	393	21.7	-155	-29.2 (-30.7, -27.7)
	20-69	8 065	126.4	6 824	106.5	-1 241	-15.7 (-15.9, -15.6)
	70+	7 431	493.5	6 361	413.5	-1 070	-16.2 (-16.4, -16.1)
	**All ages**	**16 305**	**158.6**	**13 776**	**133.1**	**-2 529**	**-16.1 (-16.2, -16)**
Necrotizing fasciitis	20-69	141	2.2	99	1.5	-42	-30.1 (-33.5, -27)
	70+	94	6.2	64	4.2	-30	-33.4 (-38.3, -29)
	**All ages**	**243**	**2.4**	**166**	**1.6**	**-77**	**-32.2 (-35.1, -29.5)**
Blood stream infections	0-4	147	24.4	116	19.4	-31	-20.5 (-22.3, -18.8)
	5-19	131	7.3	119	6.6	-12	-10.3 (-10.9, -9.7)
	20-69	3 200	50.1	2 520	39.3	-680	-21.6 (-21.9, -21.2)
	70+	8 024	532.9	7 031	457.1	-993	-14.2 (-14.3, -14.1)
	**All ages**	**11 498**	**111.9**	**9 785**	**94.5**	**-1 713**	**-15.5 (-15.6, -15.4)**
Febrile urinary tract infections	0-4	2 657	440.6	2 559	427.6	-98	-2.9 (-3, -2.9)
	5-19	1 812	101.3	1 620	89.5	-192	-11.7 (-11.9, -11.5)
	20-69	7 142	111.9	6 364	99.3	-778	-11.2 (-11.3, -11.1)
	70+	7 613	505.6	7 471	485.7	-142	-3.9 (-3.9, -3.9)
	**All ages**	**19 224**	**187.0**	**18 014**	**174.0**	**-1 210**	**-7 (-7, -6.9)**

Data on prescribed and dispensed drugs were obtained from the Swedish Prescribed Drug Register. This register contains data on all drugs dispensed in pharmacies in Sweden with a unique identification number for all citizens. Furthermore, pharmacies must submit additional information to the Swedish eHealth Agency when a prescribed drug is dispensed (i.e. place of residence, sales date of drug, ATC-code). The Swedish eHealth Agency in turn submits information on individual prescription dispensions to the National Board of Health and Welfare. The quality of the register is overall good and the risk of errors is small because prescriptions, with few exceptions, are digital and the collection process is largely automated and is based on administrative systems.

The antibiotics sold on prescriptions were categorised into groups of antibiotics commonly used to treat respiratory tract infections, urinary tract infections, and skin and soft tissue infections (Supplementary Table S1). This categorisation is according to Swedish guidelines for the treatment of infections in primary health care released through collaboration between the Medical Product Agency, Strama, and the Public Health Agency of Sweden. Data are presented as the number of persons per 100,000 inhabitants with at least one dispensed antibiotic per month for different age groups.

Information on the number of health care events due to selected infection diagnoses was obtained from the National Patient Register. This register includes all hospital admissions, doctors’ visits to outpatient specialised care (including emergency departments, day surgery, and psychiatric care) from both private and public health care providers. In outpatient specialised care, visits to hospital emergency departments account for 68% of all visits, 9% are visits to other emergency services in outpatient care, and 23% are visits to non-emergency specialised outpatient care, such as ENT clinics. Diagnoses were classified according to the international classification of diseases (ICD-10) and were categorised into nine diagnostic groups (Supplementary Table S2).

The incidence of infections per year and month was calculated for each age and diagnostic group and was defined as the proportion of persons with at least one visit to outpatient specialised care or admission to hospital in the average population during a given time period (month or calendar year) for each age group using Statistics Sweden’s population estimates. All statistical analyses were performed in SAS version 9.4.

All data were obtained from registers kept by the National Board of Health and Welfare, which is a governmental agency. According to Swedish law the population registers may be used to follow up and analyse health and quality of Swedish health care, hence no ethical approval was needed for analysis of anonymized data.

## Results

### Dispensing of prescribed antibiotics

In 2019, the number of dispensations of antibiotics commonly used to treat respiratory tract infections was 9746 per 100,000 persons (95% CI: 9728, 9764) which decreased by 28% to 7043 per 100,000 persons (95% CI 7027, 7058) in 2020.

For antibiotics commonly used to treat skin- and soft tissue infections the number of dispensions in 2019 was 3348 per 100,000 persons (95% CI: 3337, 3359) which decreased 11% to 2965 per 100,000 persons (95% CI: 2954, 2975) in 2020. For urinary tract antibiotics, the number of dispensions in 2019 was 4844 per 100,000 persons (95% CI: 4831, 4857) which decreased by 6% to 4576 per 100,000 persons (95% CI: 4563, 4589) in 2020. Overall, antibiotic dispensions decreased 2019–2020 by 18%, from 16016 (95% CI: 15993, 16038) to 13074 (95% CI: 13054, 13095) persons per 100,000 for all antibiotic groups.

**Fig. 1:**
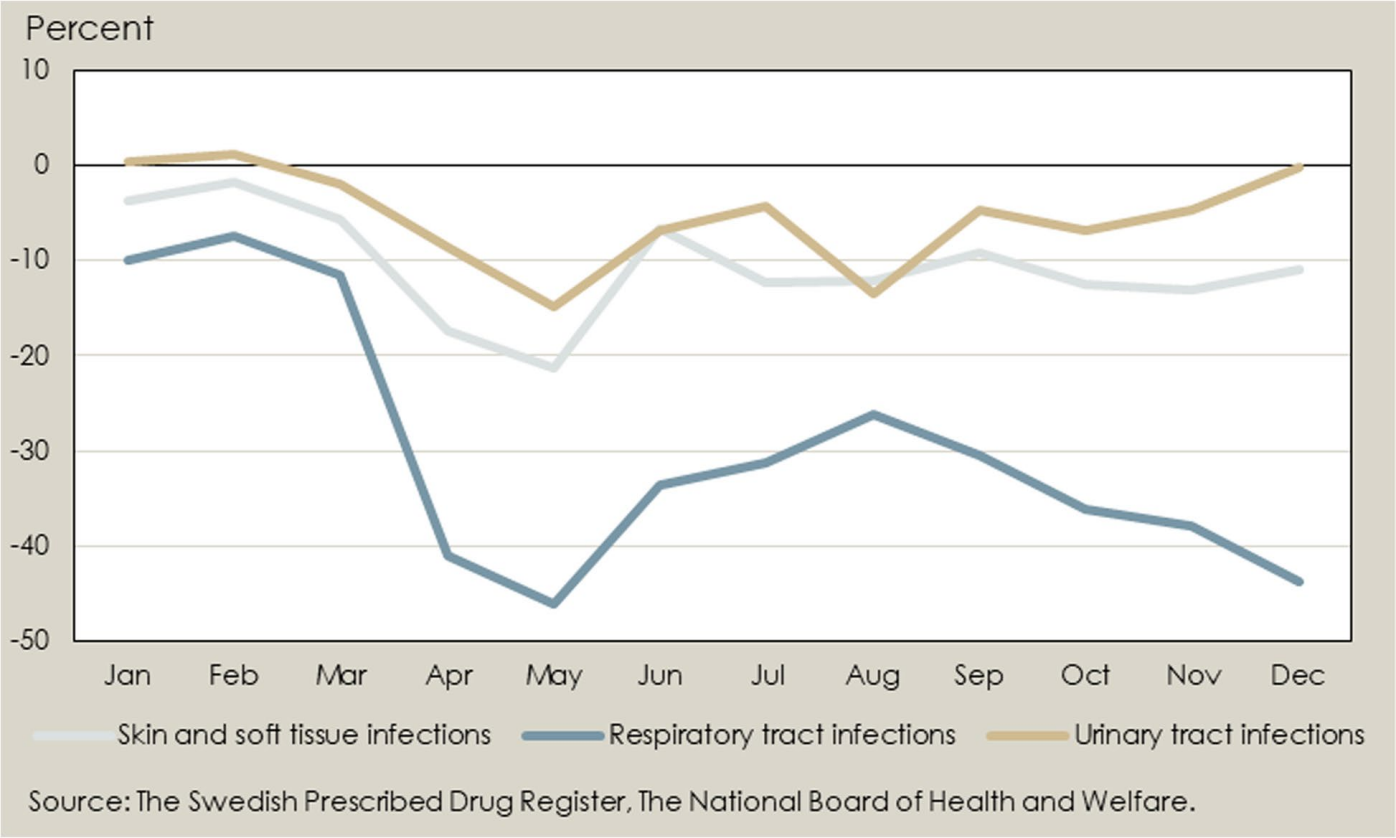
Percent change in the number of persons collecting prescriptions of different groups of antibiotics January – December 2020 compared to base-line January – December 2019. (For grouping of antibiotics, see Supplementary Table S1)

The relative difference in the number of persons collecting antibiotic prescriptions in 2020 compared to the previous year is illustrated in Fig. 1. The sales of all the three studied groups of antibiotics decreased from 2019 to 2020, but this was especially pronounced for antibiotics used to treat respiratory tract infections.

**Fig. 2:**
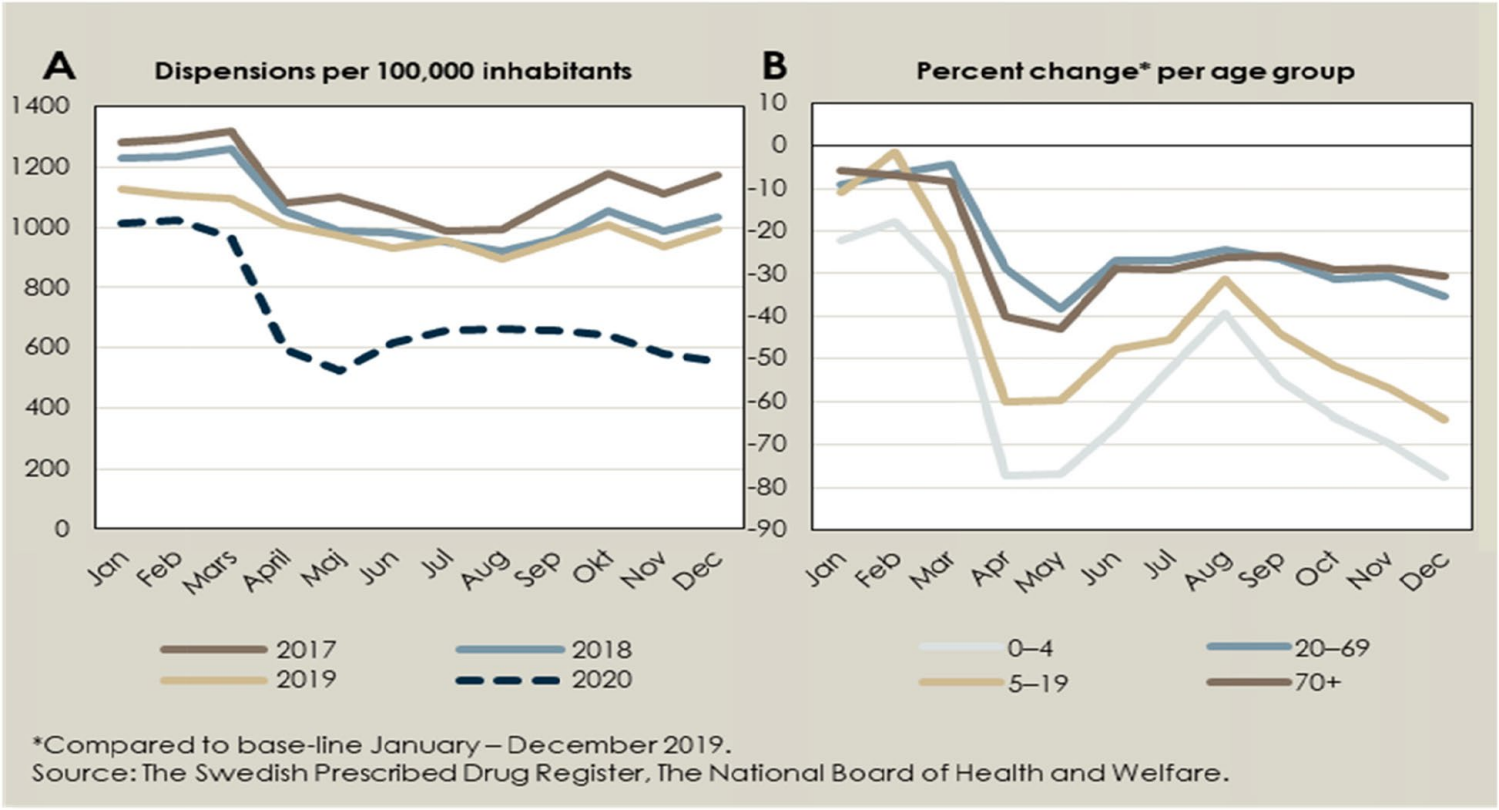
Change over time in the dispensing of antibiotics used to treat respiratory tract infections

The total number of dispensed antibiotic prescriptions decreased by 17% during 2020 compared to 2019. The number of dispensed prescriptions for treatment of respiratory tract infections decreased by 28.1% (4–44% depending on the month). For treatment of urinary tract infections in women between the ages of 18 and 79, the number of dispensed prescriptions decreased by 4.9%, and for treatment of skin-and soft tissue infections it decreased by 10.2%. As demonstrated in Fig. 2A (and Supplementary Fig. S3), the dispensing has been continuously decreasing over time, but accelerated in 2020. Fig. 2B shows that the decrease was especially large in the younger age groups of 0–4 years (–78%) and 5–19 years (–60%).

### Hospitalizations and visits to specialist care for complicating severe infections

**Fig. 3:**
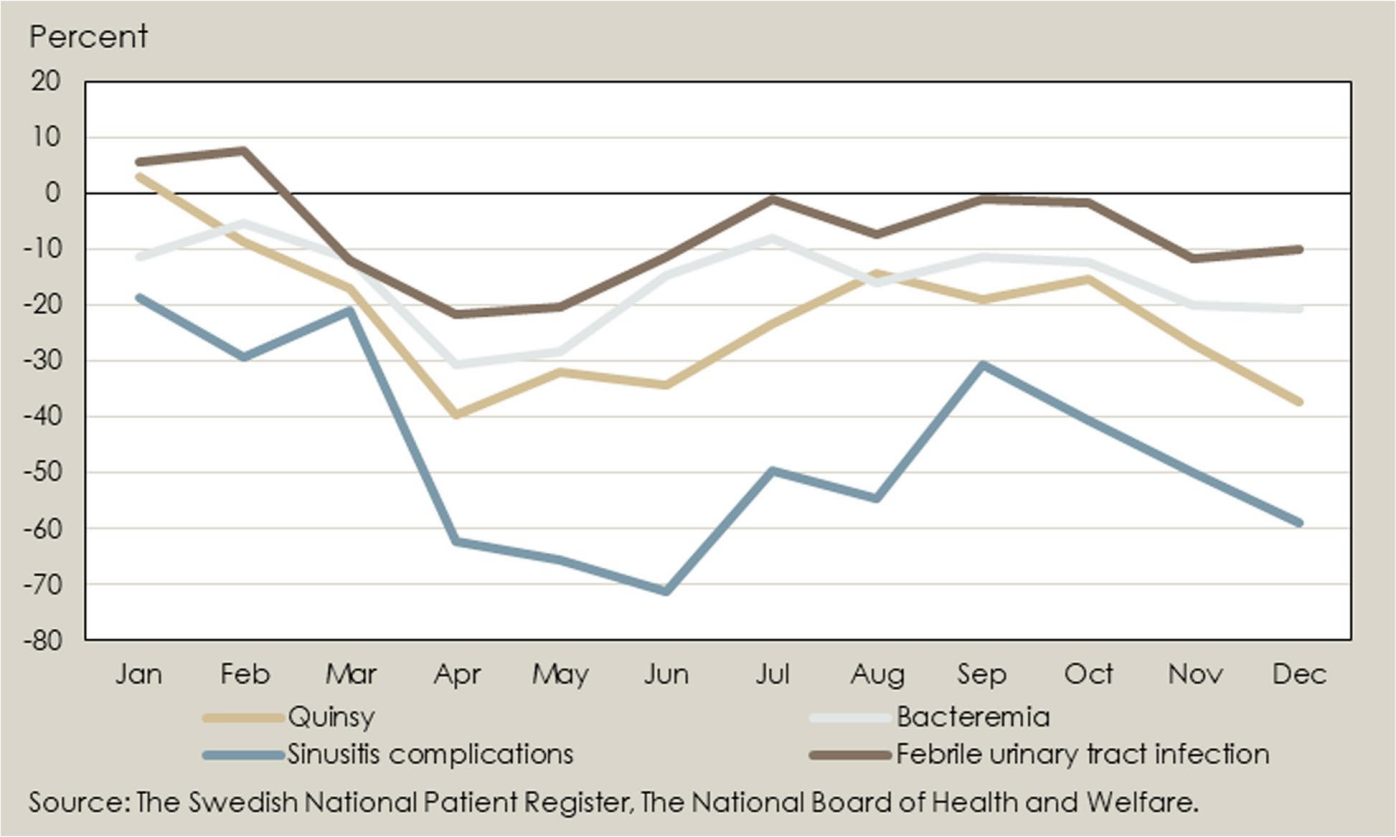
Percent change in the number of patients admitted to hospital or seeking specialised care for different diagnostic groups* January – December 2020 compared to baseline January – December 2019

**Fig. 4:**
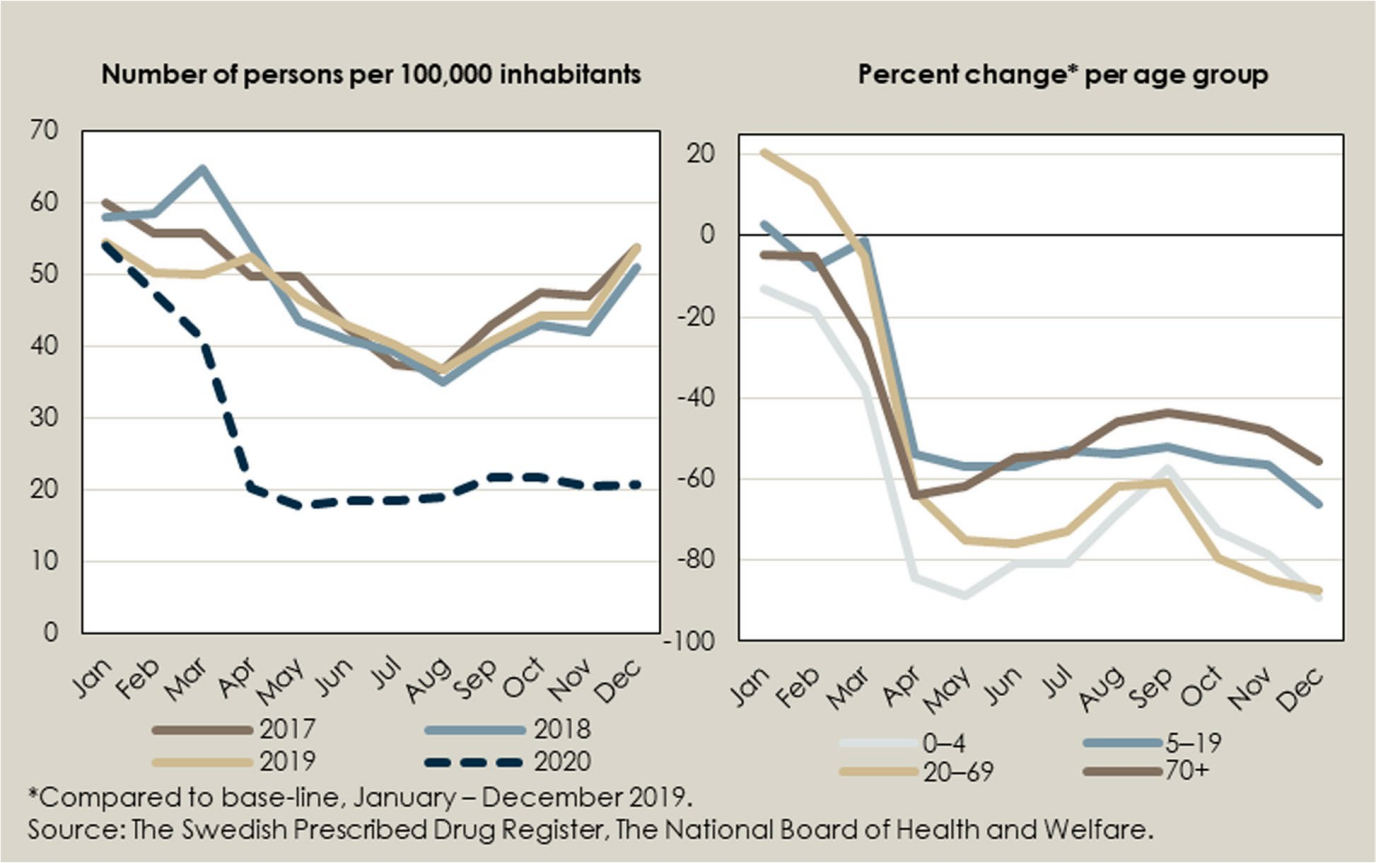
Change over time in the number of persons diagnosed with pneumonia

No increase was seen in 2020 compared to 2019 in complications from possibly untreated upper respiratory tract infections, such as quinsy after tonsillitis, mastoiditis after otitis, or meningitis/brain abscess following sinusitis (Table 1 and Fig. 3). Instead, there was a slight decrease in these diagnoses. We also studied common infections that are often treated in-hospital, including skin and soft tissue infections, pneumonia, blood stream infections, and urinary tract infections. No increase could be seen for any of these infections. For pneumonia, a pronounced decrease was seen in all age groups, from 486 events per 100,000 to 284 (–41.6%) (Fig. 4).

## Discussion

We found a sharp decrease in prescribed and dispensed antibiotics in Sweden for the year 2020 compared to 2019. Most of the decrease was attributable to a lower dispensing of antibiotic prescriptions from pharmacies for respiratory tract infections. Children aged 0–4 years had the biggest reduction with an almost 80% decrease in 2020. At the same time, we found no increase in the number of hospitalisations or specialist consultations due to complications possibly related to untreated upper or lower respiratory tract infections, skin and soft tissue infections, or urinary tract infections during the same time period.

A register-based UK study showed that antibiotic prescriptions declined along with a concomitant substantial decline in consultations for respiratory and urinary tract infections, which was most evident among those of younger ages [8]. The same was seen in a study in the US where the number of dispensed antibiotic prescriptions decreased by 39% in April 2020 and by 42% in May 2020 compared to the same months in 2017–2019 and was greatest among children ≤19 years [1]. These studies, however, did not evaluate the impact on outcome.

We did not see any increase in the number of hospitalisations or consultations for complicating severe infections in 2020. Instead, there was a decrease in the number of patients diagnosed with the studied complicating infections. In particular, the number of patients hospitalised or treated in specialised outpatient care for pneumonia decreased from around 50 per month during 2017–2019 per 100,000 to around 26 per 100,000 in 2020 (Fig. 4). The decrease was most prominent in the age group over 70 years. Similarly, a recent study using the Danish National Patient Registry also found a decrease in the incidence of pneumonia during the Covid-19 pandemic [9]. However, our data did not allow for such an analysis.

We believe that the recommendations put in place since March 2020 to mitigate the Covid-19 pandemic – namely to stay at home if you have symptoms, wash your hands, and maintain social distancing – most likely had an impact on the transmission of common infections, and thus fewer doctors’ consultations and dispensed prescriptions. For example, reported cases of seasonal influenza [10], respiratory syncytial virus [11], and norovirus [12] were all significantly fewer in 2019/2020 than in earlier years. A general reduction in opportunities for transmission is supported by the fact that the total number of days disbursed to parents staying at home caring for their sick children (rather than sending them to school with mild symtoms) increased in Sweden by 24% in 2020 compared to the previous year (Supplementary Fig. S6) [13]. Furthermore, movement data from mobile phone companies have shown that people have stayed at home more than usual, thereby reducing the number of contacts with others [14]. Before the pandemic, there was a higher transmission of respiratory viruses in the community and more people visited primary care for RTI, and probably received antibiotics inappropriately.

In accordance with this, national Swedish data on physical visits to primary care demonstrated a total decrease of 31% for the first months of the pandemic [4], and data from the Stockholm region showed a 25% decline in visits compared to the previous three years (remote consultations included) [Thomas Loogna, Controller, Region Stockholm, personal communication, May 2021]. A systematic review [3] showed that there was an overall decrease of 37% in visits to health care during the first months of the pandemic. However, another study performed in the UK found that the number of remote consultations increased from April onwards and that the total number of consultations almost normalised towards the end of 2020, while the prescriptions of antibiotics remained low [15]. In a UK study, the number of face-to-face appointments decreased in April to August of 2020 compared to the same period in 2019, while telephone appointments increased by 20.8%. This resulted in a 15.5% reduction in prescriptions, but a higher prescription rate given the number of consultations [2]. A proposed explanation for the decline in prescriptions is the combination of an increase in remote consultations and a previously high rate of inappropriate antibiotic treatment for Covid-19 during physical consultations. Our analysis included antibiotic prescriptions issued during remote consultations. Unfortunately, we could not identify how large this proportion was or how it has changed over time.

This was a population-based study where we used the national patient register to identify hospitalisations and consultations. The coverage and quality of the diagnoses in the register are high and have been validated elsewhere [16]. The Swedish prescribed drug register is unique in that it covers the entire population and measures the dispensing of prescribed drugs at an individual level (not just the number of prescriptions). Hence, we believe that our data are quite complete. The major limitation of this study is that we were unable to link consultations for respiratory and urinary tract infections and exposure to antibiotics or not with later outcomes of complicating severe infection on an individual basis. Thus, it was not possible to assess if the observed decline in complications was appropriate in relation to the decline in consultations and dispensed prescriptions. Nevertheless, this was not the objective of the study, which was to detect if an increase in complications could be seen due to the decline in dispensed prescriptions.

## Conclusions

In conclusion, we found no indications of increased incidence of complications to common infections usually treated in primary care, despite an extraordinary reduction of dispensed prescribed antibiotics. The main explanation for the decrease in dispensed antibiotic prescriptions is probably a general decreased transmission of respiratory pathogens due to changed behaviours secondary to recommendations to mitigate Covid-19 transmission. This in return led to fewer doctors’ visits, and consequently to fewer occasions to prescribe antibiotics, be they warranted or not.

## Supplementary Information

**Additional file 1.** Supplementary tables and figures

## Data Availability

The datasets generated and/or analysed during the current study are available in the links below: Swedish National Patient Register: https://www.socialstyrelsen.se/en/statistics-and-data/registers/register-information/national-patient-register/ National Prescribed Drug Register: https://www.socialstyrelsen.se/en/statistics-and-data/registers/register-information/national-prescribed-drug-register/
