# Supplementary material for: Reduced dispensing of prescribed antibiotics during the Covid-19 pandemic has not increased severe complications from common infections
Source: BMC Public Health. 2022 Feb 8;22:252. doi: 10.1186/s12889-022-12692-1 (PMC8822723; doi:10.1186/s12889-022-12692-1)

Supplementary tables and figures

The tables and figures below are supporting information alongside the article ***Reduced dispensing of prescribed antibiotics during the Covid-19 pandemic has not increased severe complications from common infections***

Figure S1. Cases of laboratory confirmed Covid-19 in Sweden 2020.


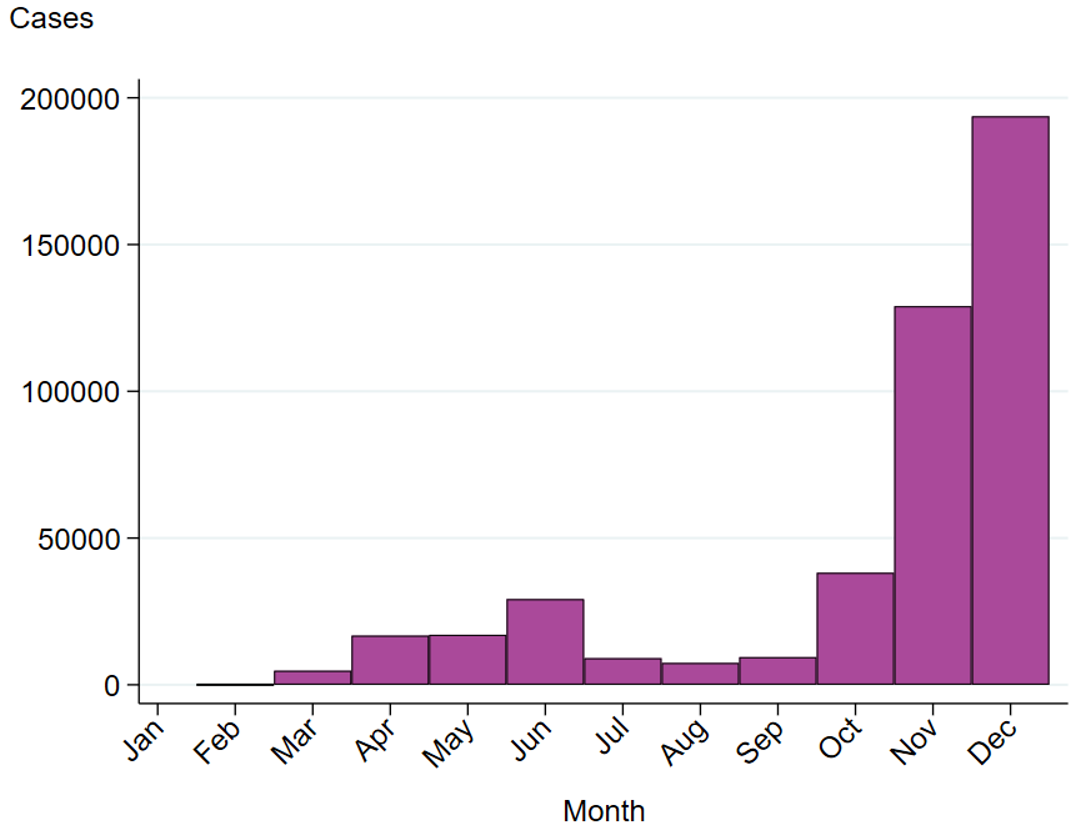


Figure S2. Cases of laboratory confirmed Covid-19 admitted to intensive care unit treatment in Sweden 2020.


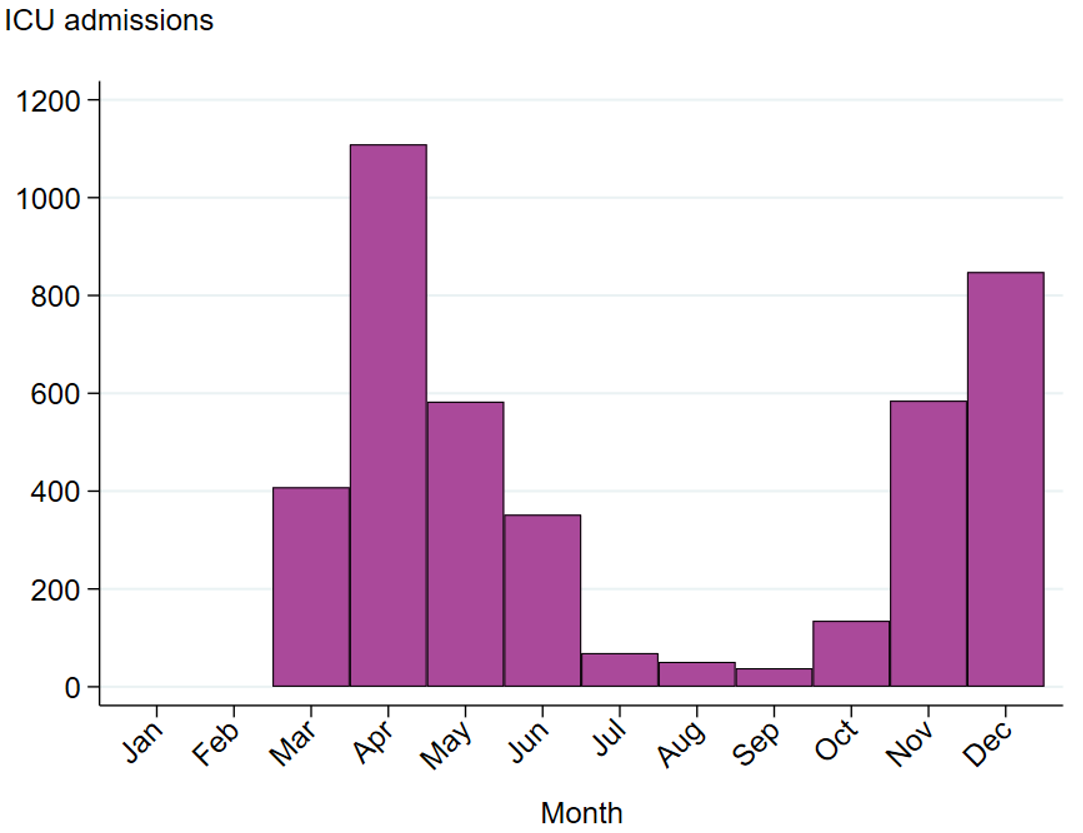


Table S1. Grouping of antibiotics according to main therapeutic indications in Swedish treatment guidelines.

| Antibiotic group | Substance name/group name | ATC-codes |
| --- | --- | --- |
| Antibiotics commonly used to treat respiratory tract infections | [Doxycycline](https://www.whocc.no/atc_ddd_index/?code=J01AA02) | J01AA02 |
|  | [Amoxicillin](https://www.whocc.no/atc_ddd_index/?code=J01CA04) | J01CA04 |
|  | [Phenoxymethylpenicillin](https://www.whocc.no/atc_ddd_index/?code=J01CE02) | J01CE02 |
|  | Amoxicillin and beta-lactamase inhibitor | J01CR02 |
|  | [Cephalosporins](https://www.whocc.no/atc_ddd_index/?code=J01DB&showdescription=no) | J01DB-DE |
|  | [Macrolides](https://www.whocc.no/atc_ddd_index/?code=J01FA&showdescription=no) | J01FA |
| Antibiotics commonly used to treat urinary tract infections | [Ciprofloxacin](https://www.whocc.no/atc_ddd_index/?code=J01MA02) | J01MA02 |
|  | [Nitrofurantoin](https://www.whocc.no/atc_ddd_index/?code=J01XE01) | J01XE01 |
|  | [Pivmecillinam](https://www.whocc.no/atc_ddd_index/?code=J01CA08) | J01CA08 |
|  | [Trimethoprim](https://www.whocc.no/atc_ddd_index/?code=J01EA01) | J01EA01 |
| Antibiotics commonly used to treat skin and soft tissue infections | Flucloxacillin | J01CF05 |
|  | [Clindamycin](https://www.whocc.no/atc_ddd_index/?code=J01FF01) | J01FF01 |

Table S2. ICD-10 codes used for the studied diagnostic groups.

| Diagnostic group | Main diagnoses | ICD-10 |
| --- | --- | --- |
| Quinsy, etc. | Peritonsillar abscess | J36 |
|  | Retropharyngeal and parapharyngeal abscess | J390 |
| Mastoiditis | Acute mastoiditis | H700 |
|  | Petrositis | H702 |
|  | Unspecified mastoiditis | H709 |
| Sinusitis complications | Acute inflammation of orbit | H050 |
|  | Acute frontal sinusitis | J011 |
|  | Acute ethmoidal sinusitis | J012 |
|  | Acute sphenoidal sinusitis | J013 |
|  | Acute pansinusitis | J014 |
| Pneumonia | Pneumonia due to Streptococcus pneumoniae | J13 |
|  | Pneumonia due to Hemophilus influenzae | J14 |
|  | Pneumonia due to Klebsiella pneumoniae | J150 |
|  | Pneumonia due to Pseudomonas | J151 |
|  | Pneumonia due to staphylococcus | J152 |
|  | Pneumonia due to streptococcus, group B | J153 |
|  | Pneumonia due to other streptococci | J154 |
|  | Pneumonia due to Escherichia coli | J155 |
|  | Pneumonia due to other aerobic Gram-negative bacteria | J156 |
|  | Pneumonia due to Mycoplasma pneumoniae | J157 |
|  | Pneumonia due to other specified bacteria | J158 |
|  | Unspecified bacterial pneumonia | J159 |
|  | Bronchopneumonia, unspecified organism | J180 |
|  | Lobar pneumonia, unspecified organism | J181 |
|  | Other pneumonia, unspecified organism | J188 |
|  | Pneumonia, unspecified organism | J189 |
|  | Pyothorax with fistula | J860 |
|  | Pyothorax without fistula | J869 |
| Meningitis and brain abscesses | Meningococcal meningitis | A390 |
|  | Hemophilus meningitis | G000 |
|  | Pneumococcal meningitis | G001 |
|  | Streptococcal meningitis | G002 |
|  | Staphylococcal meningitis | G003 |
|  | Other bacterial meningitis | G008 |
|  | Bacterial meningitis, unspecified | G009 |
|  | Intracranial abscess and granuloma | G060 |
|  | Extradural and subdural abscess, unspecified | G062 |
| Skin and soft tissue infections | Erysipelas | A46 |
|  | Cellulitis of other parts of limb | L031 |
|  | Cellulitis and acute lymphangitis of face | L032 |
|  | Cellulitis of trunk | L033 |
|  | Cellulitis and acute lymphangitis of other sites | L038 |
|  | Cellulitis and acute lymphangitis, unspecified | L039 |
| Necrotizing fasciitis | Necrotizing fasciitis | M726 |
| Febrile urinary tract infections | Acute pyelonephritis | N10 |
| Blood-stream infections | Acute meningococcemia | A392 |
|  | Meningococcemia, unspecified | A394 |
|  | Sepsis due to streptococcus, group A | A400 |
|  | Sepsis due to streptococcus, group B | A401 |
|  | Sepsis due to streptococcus group D and enterococcus | A402 |
|  | Sepsis due to Streptococcus pneumoniae | A403 |
|  | Other streptococcal sepsis | A408 |
|  | Streptococcal sepsis, unspecified | A409 |
|  | Sepsis due to Staphylococcus aureus | A410 |
|  | Sepsis due to other specified staphylococcus | A411 |
|  | Sepsis due to unspecified staphylococcus | A412 |
|  | Sepsis due to Hemophilus influenzae | A413 |
|  | Sepsis due to anaerobes | A414 |
|  | Sepsis due to other Gram-negative organisms | A415 |
|  | Sepsis, unspecified organism | A419 |
|  | Septic shock according to Sepsis-3-criteria | R572 |
|  | Systemic inflammatory response syndrome (SIRS) of non-infectious origin | R651 |

Figure S3. Overview of number of dispensed prescriptions in Sweden per 1000 inhabitants and year, 1999–2020.


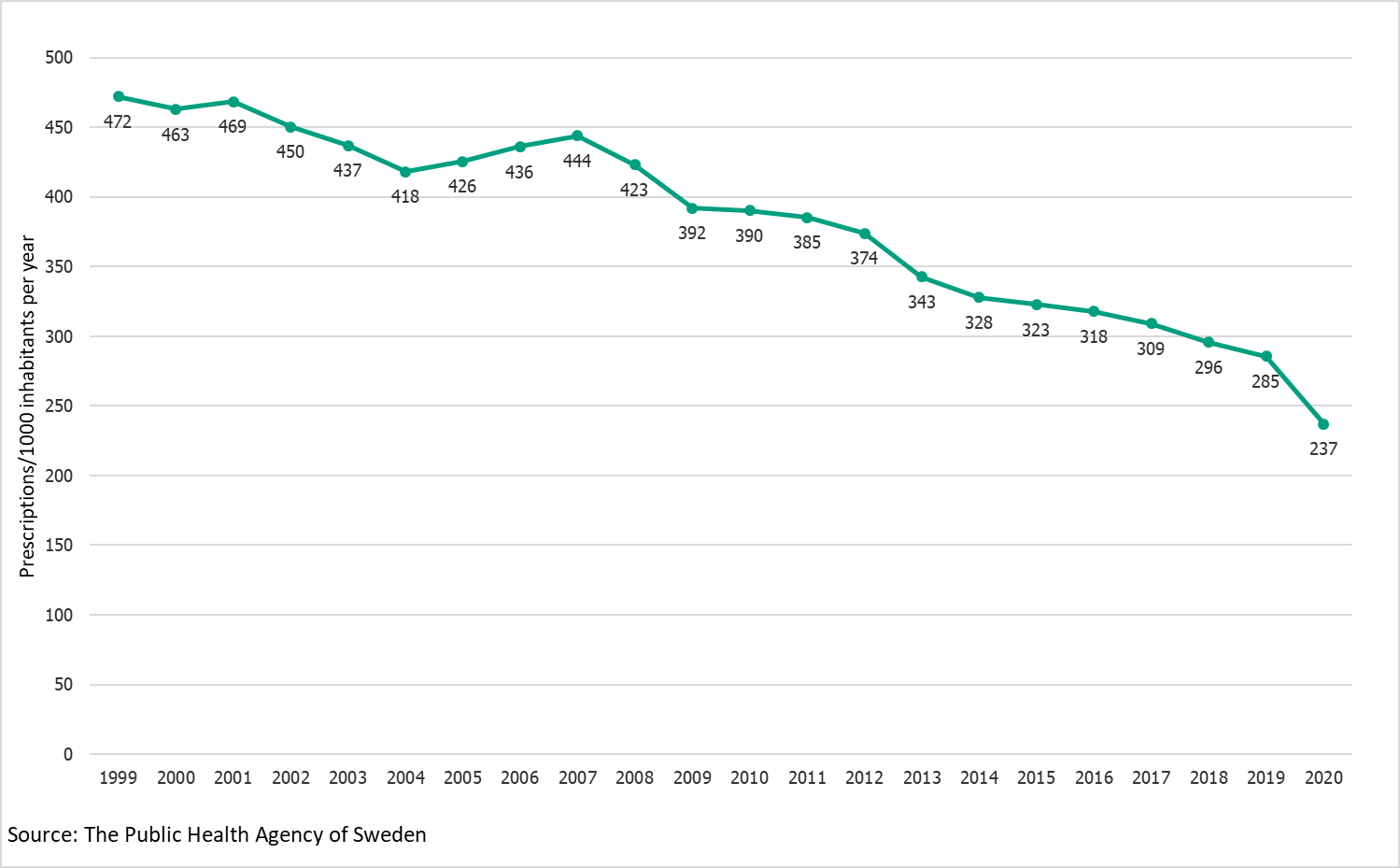


Figure S4. Change in the dispensing of prescribed antibiotics commonly used for urinary tract infections.


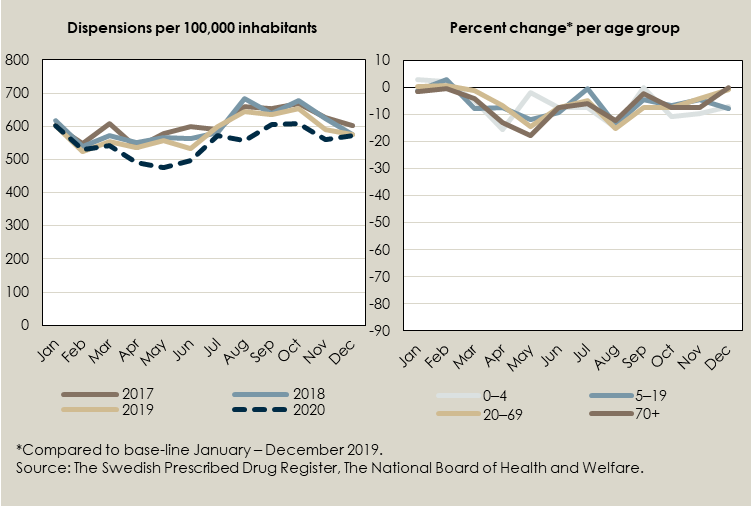


Figure S5. Change in the dispensing of prescribed antibiotics commonly used for skin and soft tissue infections.


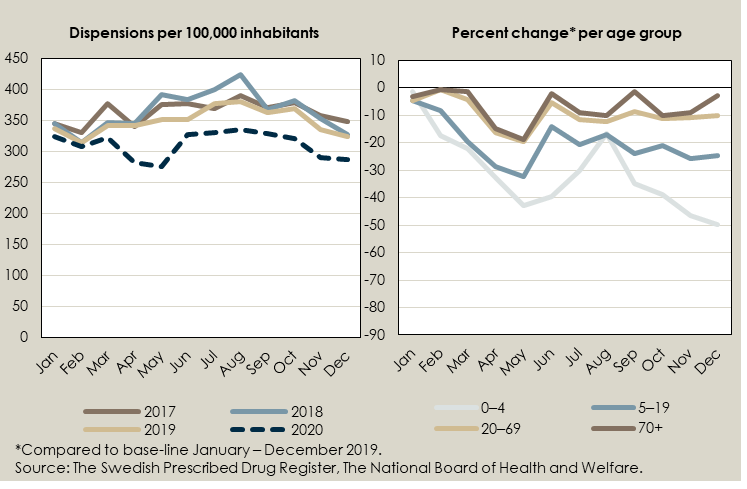


Figure S6. Number of days disbursed to parents caring for their sick children in Sweden, 2019–2020.


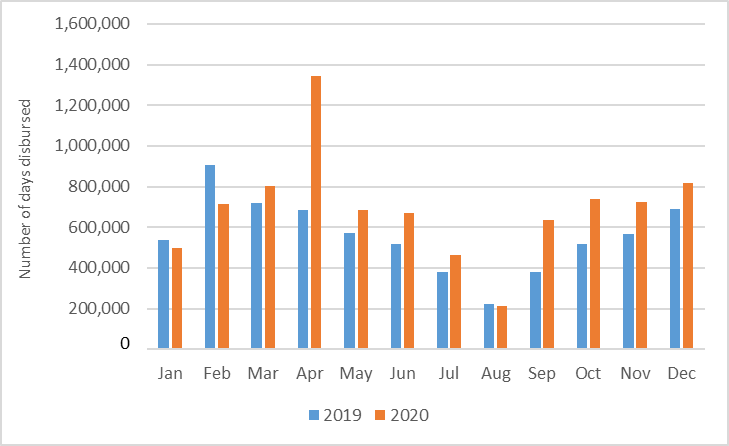

Supplement: Supplementary file 1 — Additional file 1. Supplementary tables and figures [file 12889_2022_12692_MOESM1_ESM.docx]
